# Social Cognition in Patients With Cerebellar Neurodegenerative Disorders

**DOI:** 10.3389/fnsys.2021.664223

**Published:** 2021-08-23

**Authors:** Olivera Tamaš, Milutin Kostić, Aleksandra Kačar, Elka Stefanova, Biljana Salak Ðokić, Dejana Stanisavljević, Andona Milovanović, Mirjana Ðorđević, Nenad Glumbić, Nataša Dragašević-Mišković

**Affiliations:** ^1^Neurology Clinic, Clinical Center of Serbia, Faculty of Medicine, University of Belgrade, Belgrade, Serbia; ^2^Institute of Mental Health, Faculty of Medicine, University of Belgrade, Belgrade, Serbia; ^3^Institute of Medical Informatics, Statistics and Epidemiology, Faculty of Medicine, University of Belgrade, Belgrade, Serbia; ^4^Faculty of Special Education and Rehabilitation, University of Belgrade, Belgrade, Serbia

**Keywords:** cerebellum, neurodegenerative disorder, spinocerebellar ataxia, idiopathic late-onset cerebellar ataxia, theory of mind, social cognition

## Abstract

**Objective:**

Cerebellar neurodegenerative disorders (CDs) are a heterogeneous group of disorders. It is known that the cerebellum plays a role not only in motor, but also in cognitive and social cognitive functions. The aim of this study was to investigate social cognition in patients with different CDs.

**Materials and Methods:**

Social cognition was examined in 34 patients, 12 with spinocerebellar ataxia type 1 (SCA1), 6 with spinocerebellar ataxia type 2 (SCA2), and 16 with idiopathic late onset cerebellar ataxia (ILOCA). All patients were clinically evaluated using the Scale for the Rating and Assessment of Ataxia. In addition, 34 age, sex, and education-matched healthy control (HC) subjects were similarly analyzed. Social cognition was studied using two tests: the Faux Pas Recognition Test and the Reading the Mind in the Eyes Test (RMET). An appropriate array of neuropsychological tests was used to assess the global cognitive status as well as the frontal functions and mood.

**Results:**

CD patients achieved significantly worse results on both tests of social cognition compared to the HCs. The SCA1 + 2 group achieved the poorest results on the Faux Pas Recognition Test and exhibited poor performance on all cognitive tests, but was only significantly worse compared to the ILOCA group on the Free and Cued Selective Reminding Test (FCSRT) – recognition. The patients in the SCA1 + 2 and ILOCA groups obtained similar scores on RMET. In the SCA1 + 2 group the findings significantly correlated with clinical parameters of disease severity and duration and executive functions (EFs), and with mood and executive functions in the ILOCA group. In the SCA group EFs appeared as the only significant predictor of RMET achievement. The Boston Naming Test (BTN) was a significant predictor of the CD patients’ achievement on RMET, while the BTN, the Trail Making Test Part A and FCSRT – Delayed free recall predicted their performance on the Faux Pas Recognition Test.

**Conclusion:**

Patients with CD have social cognitive impairments as demonstrated by the Faux Pas Test and the RMET test results. The SCA1 and 2 patients exhibited a more pronounced impairment compared with the ILOCA patients. The independent cognitive predictors of social cognition impairment were EFs and language.

## Introduction

Cerebellar neurodegenerative disorders (CD) encompass a group of diverse disorders affecting the cerebellum and its pathways. Some of these disorders, such as autosomal dominant cerebellar ataxias or spinocerebellar ataxias (SCAs) are characterized by cerebellar signs, as well as additional non-cerebellar signs, including extrapyramidal features, pyramidal signs, peripheral neuropathy, and in some types of SCA, cognitive deficits (Shakkottai and Fogel, 2013; Sullivan et al., 2019). SCA1 and SCA2, the two types of cerebellar ataxias which are caused by trinucleotide expansion, represent the most common types in the Serbian population (Dragasević et al., 2006). Another group of ataxias is idiopathic late-onset cerebellar ataxias (ILOCA) or sporadic adult-onset ataxias, whose etiology still remains unknown. These disorders are non-hereditary and degenerative ataxias characterized by a slowly progressive cerebellar syndrome (Barbosa et al., 2016; Klockgether, 2018).

It is a well-known and widely reported fact that the cerebellum plays a vital role in motor functions (Glickstein, 1992; Manto et al., 2012). It is equally well-known that this role also extends to cognitive and behavioral functioning (Schmahmann, 1991, 1996; Middleton and Strick, 1994; Schmahmann and Sherman, 1997; Riva and Giorgi, 2000; Ravizza et al., 2006; Frings et al., 2007; Baillieux et al., 2008; Stoodley and Schmahmann, 2009; Thompson and Steinmetz, 2009; Stoodley, 2012; Koziol et al., 2014; Hoche et al., 2018). However, scientists still disagree as to the exact role of the cerebellum in cognition (Cooper et al., 2010). Some sources have suggested that different cognitive functions are localized to specific regions of the cerebellum (Cooper et al., 2010). Patients with cerebellar disturbances have significant and relevant deficits in the working memory, visuospatial, language, and executive function (EF) domains (Ahmadian et al., 2019; Silveri, 2020). Patients with different types of hereditary ataxias, especially SCA1 and SCA2, show impairments in EF, verbal memory, and attention (Bürk et al., 2001; Le Pira et al., 2002), while ILOCA patients show impairments in visuospatial function, verbal fluency, and mental flexibility (Rentiya et al., 2018). In addition, patients with degenerative ataxias exhibit changes in emotions (Adamaszek et al., 2017) and social cognition (SC) (Sokolovsky et al., 2010).

The ability to estimate mental states of other people is a fundamental aspect of social cognition (Van Overwalle et al., 2014). Social cognition primarily implies a basic, automatic level of recognizing and attributing emotions to others by immediate perception, which can be assessed by *Reading the Mind in the Eyes Test* (RMET) (Baron-Cohen et al., 2001; Serafin and Surian, 2004; Clausi et al., 2018). In addition to this, the theory of mind (ToM) is considered as a higher level of social interference, defined as the ability to attribute mental states to others and adopting the perspectives of other persons to understand and predict behavior (Premack and Woodruff, 1978; Van Overwalle et al., 2014). According to Baron Cohen, having “theory of mind” means being able to infer a full range of mental states (beliefs, desires, intentions, imagination, emotions), or the ability to reflect on the content of one’s own and others’ minds (Baron-Cohen et al., 1999). One of the tools commonly used to assess ToM is the *Faux-Pas Recognition Test* (Stone et al., 1998; Liverta Sempio et al., 2005; Clausi et al., 2018).

The cerebellum is incorporated in associative and paralimbic circuits which are involved in social cognition (Van Overwalle et al., 2014; Heleven and Van Overwalle, 2018). Our knowledge of how exactly the neuronal circuitry supports complex human behaviors remains insufficient; however, it is known that it includes fronto-limbic connections, the medial precuneus and medial prefrontal cortex, temporoparietal junction, superior temporal sulcus (Saxe and Kanwisher, 2003; Aichhorn et al., 2009; Van Overwalle and Vandekerckhove, 2013), orbitofrontal regions, dorsal and lateral prefrontal cortex (Abu-Akel and Shamay-Tsoory, 2011), the amygdala (Adolphs, 2004), and the insula (Kipps et al., 2007). Other important participants in social cognition are mirror neurons in the ventral premotor and rostral posterior parietal cortices (Rizzolatti et al., 2006) and middle temporal gyrus (Johnstone et al., 2006).

The cerebellum and its connections have recently been added to the neural network of social cognition. It has previously been shown that not only the patients with complex cerebello-cerebral degeneration, but also those with isolated cerebellar lesions, have specific impairments in social cognition abilities (Abel et al., 2007; Sokolovsky et al., 2010; D’Agata et al., 2011; Hoche et al., 2016). Moreover, imaging studied have shown evidence of cerebellar activation during ToM tasks (Brunet et al., 2000; Calarge et al., 2003; Stoodley, 2012; Van Overwalle et al., 2014; Clausi et al., 2018; Guell et al., 2018).

The aim of this study was to assess social cognition in patients with different hereditary and non-hereditary degenerative ataxias using social cognition tests. Our hypotheses were the following: (1) Patients with hereditary and non-hereditary cerebellar disorders would exhibit impairments on the *Faux Pas Recognition Test* and RMET compared with healthy controls. (2) Patients with SCA1 and SCA2 would exhibit a more pronounced impairment on the *Faux Pas Recognition Test* and RMET compared with ILOCA patients. (3) Patients with SCA1/SCA2 would exhibit different neuropsychological profiles to those with ILOCA. (4) Executive dysfunctions would be correlated with social cognition in both groups. (5) Executive dysfunctions would be a significant predictor of social cognition in both groups of patients.

## Materials and Methods

### Participants

Social cognition was studied in 34 patients [mean age/SD: 48.9/11.8 (years); mean education/SD: 12.7/2.1 (years); M/F: 20/14]: 18 with spinocerebellar ataxias (12 SCA1 and 6 SCA2 patients) and 16 ILOCA patients. All patients presented with diffuse cerebellar atrophy on MRI, while SCA1 and SCA2 patients further exhibited brainstem atrophy and generalized cortical atrophy, and genetic disorders were confirmed by genetic analyses. SCA1 and SCA2 patients carried a heterozygous CAG triplet expansion with 53.5 ± 5.2 repeats (range 48 to 63) in the coding ATXN1 region and CAG triplet expansion with 37.6 ± 1.5 repeats (range 36 to 40) in the coding ATXN2 region, respectively. The subjects were diagnosed and tested at the Neurology Clinic of the Clinical Center of Serbia (CCS) in 2017–2020. The patients were taking dietary supplements (vitamin E and selenium, Coenzyme Q10). None of the patients showed any major psychiatric disorders, anxiety or depressive traits, nor were they taking antidepressants or any other psychoactive drugs. In addition to the previously described body of patients with ataxias, 34 age, sex, and education-matched healthy control (HC) subjects were enrolled in the study. An appropriate battery of tests was used to assess global cognitive status, frontal neuropsychological functions, social cognition, and mood. All participants were informed of the purpose, procedures, and scope of all the examination and were included in the screening only after providing their written informed consent for participation.

### Clinical Assessment

The patients were clinically evaluated using SARA (*Scale for the Rating and Assessment of Ataxia)*, which assesses a range of different impairments in cerebellar ataxia (Schmitz-Hübsch et al., 2006). This Scale assesses the following aspects: truncal ataxia, speech disturbance and limb ataxia. It has eight categories with an accumulative score ranging from 0 (no ataxia) to 40 (most severe ataxia).

### Social Cognition

#### Faux Pas Recognition Test

A social *faux pas* occurs when a speaker says something without considering that the listener might not want to hear it or might be hurt by what has been said, implying a false or mistaken belief (Baron-Cohen et al., 1999). In normal circumstances, people are usually able to recognize the *faux pas* and understand that the person who said something inappropriate did not know that that the listener would be upset by the *faux pas*. To understand *faux pas* the subject must identify wrong behavior in the context of predicted social interactions. The events that constitute a *faux pas* are unpredicted, and the subject needs to compare events and social expectations constantly while in no-*faux pas* stories the events are expected and require a low level of prediction.

We used a Serbian adaptation of the revised version of the *faux pas* stories (Baron-Cohen et al., 1999; orđević, 2013) to evaluate the ability to recognize a social *faux pas*. The detection of a *faux pas* requires both a cognitive understanding of false or mistaken beliefs and an appreciation of the emotional impact of a statement on the listener. Twenty stories were read to the participants, who were provided with copies of the stories to read along as the stories were read and check back over their contents. Ten of the stories involved a social *faux pas* (*Faux Pas* stories), and 10 were control stories in which no *faux pas* occurred (No-*Faux Pas* stories). After listening to each story, the subject was asked whether anyone had said anything they should not have said. When a *faux pas* was identified, specific clarifying questions were asked to verify the participant’s understanding of the mental and emotional state of the agent involved in the stories. Samples of *Faux Pas* and No-*Faux Pas* stories with respective questions are reported. Separate reports were made for the: (a) *faux pas*-related questions about the stories containing a *faux pas*, (b) control questions on the faux *pas* stories, (c) *faux-pas*-related questions on the control stories, and (d) control questions on the control stories. Separate reporting helped keep track of the type of errors the participants had made i.e., whether they had more *faux-pas*-related errors (ToM errors) or errors on the factual control questions. If a control question was answered incorrectly, other errors made in connection with that particular story were interpreted with caution. Each correctly answered question related to a *Faux Pas* story was scored as 1, yielding a maximum score of 6 for each correctly identified *Faux Pas* story and altogether 60 for the total of 10 *Faux Pas* stories. For each No-*Faux Pas* story a score of 2 was given if the subject correctly identified the absence of a *faux pas*. Two questions assessing the comprehension of the story material were asked after each story. The results were converted into percentages.

#### Emotion Recognition Test

To examine the participants’ ability to infer the mental states of others, we used a Serbian adaptation of the revised version of the *Reading the Mind in the Eyes Test* (RMET) (Baron-Cohen et al., 2001; orđević et al., 2017). The test evaluates the first stage (automatic) mentalizing and consists of 36 photographs of the actors’ eye region, each printed on a separate sheet of paper. Four adjectives corresponding to complex internal mental state descriptors were given.

The participants were required to identify the sex of the people in the pictures. They were also asked to decide which of the four words best described what the individuals in the photographs were thinking or feeling. Only one of the four words (the target word) correctly described the mental state of the person in the photograph. Although more than one word might have seemed applicable, the participants were instructed to choose only one, making sure to carefully read all four before making a decision. The answers were not timed. However, the participants were asked to complete the task as quickly as possible. The use of a dictionary was allowed in case of an unfamiliar word. For each correct answer, the participants received 1 point. The maximum number of points was 36. The test results were then divided by the maximum number of points (36) and converted into percentages.

#### Neuropsychological Screening

Cognitive functions were assessed using selected tests from a neuropsychological battery that included the following:

Global cognitive functioning was measured with the *Addenbrooke’s Cognitive Examination –*

*Revised* (ACE-R) (Mioshi et al., 2006). The test consists of 5 subscales designed to assess attention and orientation, fluency, language, visuospatial skills, and verbal memory. For the assessment of global functioning we used the total score. To test this aspect we also used the standard *Mini-Mental State Examination* (MMSE) (Folstein et al., 1975).

Learning and episodic verbal memory were assessed with the *Free and Cued Selective Reminding Test* (FCSRT) (Buschke, 1984; Grober et al., 1988) using the immediate total free recall (total number of items retrieved over 3 learning trials), delayed free recall (number of words freely recalled after a 30-min delay), and recognition (number of recognized words after delayed free and cued recall).

Orientation and attention were tested with the help of several subtests: the *Orientation and attention* subscale from the ACE-R; the *Digit Span*-subtest from the VITI (*Vekslerov Individualni Test Inteligencije*) (Pavlović, 2003), a Serbian adaptation of *Wechsler’s Adult Intelligence Scale – Revised*. The subjects were asked to repeat auditorily presented sequences of digits forward (up to 9 digits) and backward (up to 8 digits), and trials were administered until two failures of each span length. The total number of correct answers after the transformation into a scaled score was then used. To assess attention and psychomotor speed, we used the *Trail Making Test Part A* (TMT-A), a timed measure of selective attention to visually presented information (Lezak, 1995). The TMT-A consists of 25 circles distributed over a sheet of paper. The patients were asked to draw lines to connect them in ascending order as fast as possible.

Calculation was assessed using the *Arithmetics*-subtest from VITI (Pavlović, 2003), which consists of a series of verbally presented arithmetic questions of increasing difficulty. Transformed total scores were used.

Confrontation naming was assessed with the *Boston Naming Test* (BNT) using a raw score that represents the total of correctly named objects (spontaneously or after giving a semantic cue) (Kaplan et al., 1983).

Visuospatial processing was tested using the *Hooper Visual Organization Test* (HVOT) (Western Psychological Services, 1983). The total number of correct answers was recorded.

Executive functions were evaluated using several subtests: the *Stroop Color and Word Test (SCWT)* (Lezak, 1995). This test involves an interference task, requiring the naming of color ink, preventing the reading of words for which the color and meaning of the words are incongruent (for example, the word “red” written in green). The analysis includes the time (in seconds) required to complete the task. Verbal working memory was assessed using the *Digit Ordering Test* (DOT) (Cooper et al., 1991): a series of seven digits that has to be memorized and immediately recalled in ascending order. The total number of correct answers and the maximum forward range were used in the analysis. Letter fluency (number of words beginning with the letters S, K, and L produced in 60 s) and category fluency (number of animals produced in 60 s) (Lezak, 1995) were used as measures of verbal divergent thinking. The analysis included the total raw score for all three letters, the score for category fluency, and the corrected values for both of these measures. All tests were conducted by a qualified neuropsychologist in a standardized manner consistent across subjects.

### Mood Assessment

Mood was evaluated with the help of two tests:

### Depression Assessment

The *Hamilton Depression Rating Scale* (HDRS) (Hamilton, 1960), which consists of 17 questions, was used to assess the patients’ levels of depression. The assessment, which was in the form of a structured interview, was conducted by a trained physician. Total scores vary from 0 to 53. For the HDRS, a score of 0–7 is generally accepted as being within the normal range, while a score of 20 or higher indicates at least moderate severity.

### Anxiety Assessment

The *Hamilton Anxiety Rating Scale* (HARS) (Hamilton, 1959) was used to determine the presence of anxiety in our patients. The Scale is completed by a trained physician acting as the examiner, based on interviews held with patients. The Scale consists of 14 items which are individually rated from 0 (not present) to 4 (severe). Total scores vary from 0 to 56. Patients with score ≥13 were considered anxious.

### Data Analysis

The assumption of normality of dependent variables (*Faux Pas Recognition Test*, RMET, neuropsychological tests, the duration of the disease, SARA, mood assessment and EF, and other neuropsychological functions tests) was determined based on the results of the *Kolmogorov-Smirnov* and *Shapiro-Wilk* tests, which indicate the application of non-parametric techniques (*p* < 0.05). The non-parametric *Kruskal Wallis Test*, with *post hoc* pairwise comparisons, was used to compare the three groups (SCA, ILOCA, and HC) in terms of socio-demographic variables, mood assessment tests, *Faux Pas Recognition Test*, RMET and neuropsychological tests. The *Mann-Whitney U Test* for independent samples was used to detect differences in the scores between the two CD groups, on variables assessed only on these groups, such as duration of the disease, clinical characteristics/SARA. The *Spearman rank-order correlation coefficient* was used to correlate the *Faux Pas Recognition Test* and RMET results with the duration of the disease, clinical characteristics/SARA, psychiatric tests and EF tests in all three groups (SCA1 + 2, ILOCA, HC). *Multiple regression analysis* was used to determine whether EFs qualify as significant predictors on the *Faux Pas Recognition Test* and RMET in SCA and ILOCA groups. Same analysis was used to determine whether all neuropsychological tests could significantly predict scores on the *Faux Pas Recognition Test* and RMET in both CD groups. The statistical analyses were performed using IBM SPSS software 21.

## Results

Demographic details of CD patients and HC are displayed in Table 1. The *Kruskal Wallis Test* showed a significant difference in the mean age (χ^2^ = 6,961; *p* = 0.031) between the three groups. The test showed no significant difference in the mean level of education (χ^2^ = 1,702; *p* = 0.427) between the three groups. The mood assessment test results seem to be within the normal expected range for each of the three groups, and the *Kruskal Wallis Test* showed a significant difference in the mean HDRS score (χ^2^ = 29,414; *p* = 0.0001) and HARS score (χ^2^ = 20,825; *p* = 0.0001). *Post hoc* pairwise comparisons with Bonferroni correction indicated differences between:

**TABLE 1:** Socio-demographic characteristics of groups, clinical characteristics and mood scales – descriptive statistics.

	**SCA1 + 2 (*n* = 18, 10M + 8F)**		**ILOCA (*n* = 16, 10M + 6F)**		**HC (*n* = 34, 16M + 18F)**	
	**Mean/Median (sd)**	**Range**	**Mean/Median (sd)**	**Range**	**Mean/Median (sd)**	**Range**
Age (yrs)	43.78/42.5 (12.4)	22–68	54.8/55.5 (7.9)	38–68	49.1/44 (11.8)	22–68
Education (yrs)	12.2/12 (2.3)	8–16	13.1/12 (1.7)	11–16	12.7/12 (2.1)	8–16
Onset of disease (yrs)	32.7/30.5 (10.6)	16–53	47.6/48.5 (8.9)	28–62	–	–
Disease duration (yrs)	10.9/8.5 (9.1)	2–38	7.1/7.5 (4.2)	2–15	–	–
Truncal ataxia	7.4/7 (2.1)	4–12	4.4/4 (1.9)	2–8	–	–
Dysarthria	2.5/3 (0.6)	1–3	1.7/1.5 (1.1)	0–4	–	–
Limb ataxia	5.3/5 (2.2)	3–12	2.9/2.5 (1.6)	0–6	–	–
SARA^1^	15.3/15 (3.8)	10–22	9.0/8.5 (3.7)	3–18	–	–
HDRS^2^	6.7/7.5 (4.1)	0–17	7.1/7 (3.9)	1–15	1.7/0 (2.7)	0–13
HARS^3^	9.6/10.5 (5.9)	0–17	7.3/6 (6.2)	0–17	2.1/1 (3.6)	0–19

*^1^Scale for the Rating and Assessment of Ataxia (SARA), ^2^Hamilton Depression Rating Scale, ^3^Hamilton Anxiety Rating Scale. Source: Author’s calculation.*

- SCA1 + 2 and ILOCA groups in age (*t* = −26,781; *p* = 0.025),
- HC and SCA1 + 2 groups (*t* = −24,346; *p* = 0.0001) and HC and ILOCA groups (*t* = −26,735; *p* = 0.0001) in HDRS score
- HC and SCA1 + 2 groups (*t* = −17,173; *p* = 0.011) and HC and ILOCA groups (*t* = −24,346; *p* = 0.0001) in HARS score.

For variables applicable only to the CD groups (SCA1 + 2 and ILOCA), *Mann-Whitney U Tests* indicated significant differences in onset of disease (*U* = 42.5; *z* = −3.505; *p* = 0.0001), truncal ataxia (*U* = 39.5; *z* = −3.632; *p* = 0.0001), dysarthria (*U* = 78; *z* = −2.398; *p* = 0.017), limb ataxia (*U* = 47; *z* = −3.372 *p* = 0.001), and SARA (*U* = 34.5; *z* = −3.783; *p* = 0.0001) between patients in the SCA1 + 2 and ILOCA groups.

Mean values, standard deviations and range are shown in Table 1.

### Social Cognition Profile in CD Patients vs. HC

**FIGURE 1:**
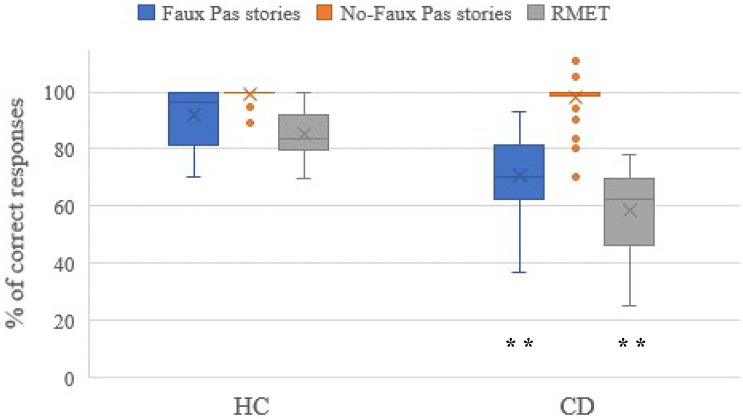
The scores of the social cognition battery. Data are presented as the percentage of the total number of correct responses for the Faux Pas stories (max = 60) and the No-Faux Pas stories (max = 20), and for the RMET (max = 36). The mean scores of the accuracy percentage, where 0% is totally wrong and 100% is totally correct, are reported for the CD and HC groups, and significant differences of p < 0.01 are indicated by ^∗∗^.

We identified significant differences in the performance on the *Faux Pas Recognition Test* and RMET between the CD patients and HCs. Box plots illustrating the differences in the average scores (in%) obtained from CD and HC subjects in each social cognition task (*Faux Pas* stories, No-*Faux Pas* stories and RMET) are shown in Figure 1. In the *Faux Pas Recognition Test* the patients with cerebellar hereditary and non-hereditary disorders showed impaired scores compared to HCs [*U* = 129.5, *z* = −5.544, *p* = 0.0001, *r* = 0.67 (high effect size, 95%CI: 0.523 to 0.788)]. Moreover, the patients with cerebellar hereditary and non-hereditary disorders obtained significantly lower scores than HCs in the RMET [*U* = 17.0, *z* = −6.895, *p* = 0.0001, *r* = 0.84 (high effect size, 95%CI: 0.756 to 0.899)].

### Differences in the Faux Pas Recognition Test and RMET Outcomes Among All Studied Groups

We further investigated the differences in the performance on the Faux Pas Recognition Test and RMET tests between groups. The average score (standard deviation) on the *Faux Pas Recognition Test* was 63.8 (11.6) for SCA1 + 2, 78.8 (13.9) for ILOCA and 91.9 (10.2) for HC. The average score (standard deviation) on RMET was 52.8 (14.4) for SCA1 + 2, 64.4 (10.4) for ILOCA and 85.3 (8.3) for HC. *Kruskal Wallis Test* showed a significant difference in the mean *Faux Pas Recognition Test* score (χ^2^ = 36,661; *p* = 0.0001) and RMET score (χ^2^ = 49,316; *p* = 0.0001). *Post hoc* pairwise comparisons with Bonferroni correction indicated differences between:

- The scores for all three groups on the *Faux Pas Recognition Test*; SCA1 + 2 and ILOCA (*t* = −16,417; *p* = 0.045), SCA1 + 2 and HC (*t* = 34,108; *p* = 0.0001), ILOCA and HC (*t* = 17,691; *p* = 0.009). Based on medians, the patients in the SCA1 + 2 group obtained lower scores than the patients in the ILOCA group, while both of these groups obtained lower scores than the HC group participants.
- CD groups in comparison to HC group on RMET scores, SCA1 + 2 and HC (*t* = 37,250; *p* = 0.0001), ILOCA and HC (*t* = 28,219; *p* = 0.0001). Based on median values, the patients in the SCA1 + 2 and ILOCA group obtained similar scores, which were lower than HC group participants.

### Neuropsychological Findings

We investigated intergroup comparisons (SCA1 + 2, ILOCA, and HC) in scores on the neuropsychological tests. Median and significance for intergroup comparisons can be seen in Table 2.

**TABLE 2:** Neuropsychological screening – descriptive statistics and group comparison.

	**SCA1+2 (*n* = 18)**		**ILOCA (*n* = 16)**		**HC (*n* = 34)**		**SCA1+2 - HC**	**ILOCA - HC**
	**Mean/Median (sd)**	**Range**	**Mean/Median (sd)**	**Range**	**Mean/Median (sd)**	**Range**	**t**	**t**
**Global cognitive functioning**								
MMSE^1^	27.2/27.5 (2.8)	19–30	27.8/27.5 (1.7)	25–30	29.1/30 (1.2)	27–30	16.428*	15.22*
ACE-R-total^2^	82.6/85.5 (12.0)	56–99	89.9/89 (4.9)	80–98	95.9/97(4.2)	86–100	29.601**	19.41**
**Learning and episodic verbal memory^3^**								
FCSRT-total free recall	20.6/20.5 (6.8)	5–33	22.1/22.5 (4.9)	15–32	29.2/34 (6.9)	8–40	23.662**	19.756**
FCSRT-delayed free recall	8.4/8.5 (2.7)	5–13	10.4/10 (2.5)	5–15	11.3/12 (2.6)	4–15	19.941**	
FCSRT-recognition	14.4/14.5 (1.7)	12–16	15.5/16 (1.5)	10–16	15.9/16 (0.2)	15–16	17.905**	
ACE-R-verbal memory	21.7/23 (4.9)	6–26	23.3/23 (2.2)	19–26	25.3/26 (1.4)	21–26	22.26**	17.774**
**Orientation and attention**								
ACE-R-orientation and attention	16.6/17 (1.8)	12–18	17.3/17.5 (0.9)	16–18	17.6/18 (0.7)	16–18		
TMT-A	83.6/74 (36.6)	30–157	49.3/42.5 (17.0)	22–82	33.4/30 (14.7)	18–78	31.183**	17.169*
Digit-span	7.7/8 (2.1)	4–11	8.3/8 (2.9)	4–13	11.3/11.5 (2.1)	7–15	24.977**	20.088**
**Calculation**								
Arithmetic-VITI^4^	9.1/9 (3.2)	3–14	11.3/11 (1.6)	9–14	13.8/14 (0.5)	12–14	32.647**	24.014**
**Language**								
ACE-R-language	23.2/25 (3.2)	15–26	24.8/25 (0.8)	24–26	25.4/26 (1.2)	22–26	17.188**	
BNT-Total	50.3/52 (6.4)	40–58	53.3/54 (3.1)	45–57	54.4/55 (3.9)	40–59		
**Visuospatial processing**								
HVOT^5^	17.2/17 (6.3)	8–25	21.3/21.5 (3.1)	16–25	24.6/25 (2.9)	17–29	26.103**	18.39**
ACE-R-visuospatial skills	13.2/14 (3.1)	8–16	15.3/16 (1.0)	13–16	15.3/16 (1.4)	11–16	14.634**	
**Executive functions**								
SCWT^6^	91.6/90 (37.6)	45–154	97.3/98 (41.2)	36–180	57.9/53.5 (21.5)	10–98	18.088**	19.488**
DOT Total^7^	5.3/5 (1.9)	2–9	6.0/6 (1.3)	4–9	7.9/8 (2.1)	4–12	23.5**	18.188**
ACE-R-phonemic fluency	3.8/4 (1.6)	1–7	4.4/4 (1.5)	2–7	5.7/6 (0.9)	4–7	23.034**	17.086*

^1^Mini Mental State Examination, ^2^Addenbrooke’s Cognitive Examination – Revised, ^3^Free and Cued Selective Reminding Test, ^4^Serbian adaptation of the Wechsler Adult Intelligence Scale – Revised, ^5^Hooper Visual Organization Test, ^6^Stroop Color and Word Test, ^7^Digit Ordering Test. Source: Author’s calculation (** indicate *p* < 0.01, while * indicates *p* < 0.05).

The *Kruskal Wallis Test* showed a significant difference in the mean MMSE score (χ^2^ = 11,299; *p* = 0.004), ACE-R – total (χ^2^ = 27,801; *p* = 0.0001), FCSRT – total free recall (χ^2^ = 21,097; *p* = 0.0001), FCSRT – delayed free recall (χ^2^ = 12,177; *p* = 0.002), FCSRT – recognition (χ^2^ = 18,528; *p* = 0.0001), ACE-R – verbal memory (χ^2^ = 19,121; *p* = 0.0001), TMT-A (χ^2^ = 30,569; *p* = 0.0001), Digit Span (χ^2^ = 23,136; *p* = 0.0001), Arithmetic-VITI (χ^2^ = 41,979; *p* = 0.0001), ACE-R – phonemic fluency (χ^2^ = 19,495; *p* = 0.0001), HVOT (χ^2^ = 23,541; *p* = 0.0001), SCWT (χ^2^ = 15,779; *p* = 0.0001), ACE-R-Language (χ^2^ = 11,356; *p* = 0.003), ACE-R-visuospatial skills (χ^2^ = 8,273; *p* = 0.016), DOT Total (χ^2^ = 20,185; *p* = 0.0001).

*Post hoc* pairwise comparisons with Bonferroni correction indicated differences between both CD groups in comparison to the HC group on:

- MMSE score, ACE-R – total, FCSRT – total free recall, ACE-R – verbal memory, TMT-A, Digit Span, Arithmetic-VITI, ACE-R – phonemic fluency, HVOT, SCWT, DOT Total. Based on median values, the patients in SCA1 + 2 and ILOCA group obtained similar scores, and worse than HC group participants.

On some scales, *post hoc* pairwise comparisons with Bonferroni correction indicated differences only between SCA1 + 2 and HC group:

- FCSRT – delayed free recall, ACE-R-Language, ACE-R-visuospatial skills. Based on medians, the patients in SCA1 + 2 group obtained worse scores than HC group participants, while patients in the ILOCA group obtained scores between the other two.

And lastly, on FCSRT – recognition scale, *post hoc* pairwise comparisons with Bonferroni correction indicated differences between the SCA1 + 2 and HC groups (*t* = 17,905; *p* = 0.0001), and between the SCA1 + 2 and ILOCA groups (*t* = −13,424; *p* = 0.019). Median values indicate that the patients in SCA1 + 2 group obtained lower scores than the other two groups, patients in the ILOCA group and HC participants.

### Associations of the Social Cognitive Profile With Sociodemographic, Clinical, and Genetic Features of CD Patients With Their Neuropsychological Findings and Mood State

The duration of the disease (*r* = −0.555, 95%CI: −0.119 to −0.811, *p* = 0.017), truncal ataxia (*r* = −0.557, 95%CI: −0.122 to −0.812, *p* = 0.016) and SARA score (*r* = −0.561, 95%CI: −0.128 to −0.814, *p* = 0.015) showed a significant inverse (negative) correlation with the *Faux Pas Recognition Test* in the SCA1 and SCA2 groups of patients, while the *SCWT* (*r* = −0.482, 95%CI: −0.002 to −0.781, *p* = 0.050) correlated inversely with RMET. In the ILOCA group of patients, RMET correlated with HDRS (*r* = 0.498, 95%CI: 0.004 to 0.796, *p* = 0.050) and HARS (*r* = 0.541, 95%CI: 0.062 to 0.817, *p* = 0.030) and *ACE-Phonemic Fluency* (*r* = 0.545, 95%CI: 0.068 to 0.819, *p* = 0.029). There was no significant correlation in the SCA1 and SCA2 groups of patients between the number of repeats in the expanded allele and the achievement on *Faux Pas Recognition Test* and RMET.

### Predictors of Social Cognition Impairment in CD Patients

The final goal of this study was to determine whether EFs are significant predictors of performance in social cognition tests in SCA1 + 2 and ILOCA patients. The predictor variables (SCWT, DOT Total, ACE-R-Phonemic Fluency) did not show significant prediction of the *Faux Pas Recognition Test* scores in the SCA1 + 2 group (*F* = 1.802; df = 3.13; *p* > 0.05), nor in the ILOCA group (*F* = 1.269; df = 3.11; *p* > 0.05). In addition, in the ILOCA group, EFs (SCWT, DOT Total, ACE-R-Phonemic Fluency) did not show significant prediction of RMET (*F* = 1.95; df = 3.11; *p* > 0.05). On the other hand, in the SCA1 + 2 group, EFs appeared as significant predictors of the *Emotion Recognition Test* (*F* = 4.05; df = 3.13; *p* = 0.031), explaining 48.3% of the variance. The only significant predictor was *SCWT* (β = −0.631, 95% CI from −1.04 to −0.22, *t* = −3.006, *p* < 0.05). If the *SCWT* score were to increase by one standard deviation, the achievement scores on the RMET would likely decrease by 0.63 units of standard deviation in the patients of the SCA1 + 2 group. Since only one EF test could predict social cognition impairment, we wanted to determine which other domains of neuropsychological tests might be significant predictors of failure in social cognition in both groups of CD patients.

First, we entered all neuropsychological tests in the regression model, but some of them showed suppression effects that needed to be removed from the model. The final list of neuropsychological predictors (MMSE score, FCSRT – Delayed free recall, TMT-A, BNT – Total, SCWT and DOT Total) showed significant prediction of *Faux Pas Recognition Test* (*F* = 7.458; df = 6.23; *p* = 0.0001) in CD patients, explaining 66% of the variance. Standardized beta coefficients show that BNT – Total (β = 0.47, 95%CI from 0.16 to 0.77, *t* = 2.97, *p* = 0.007) has the greatest effect, followed by TMT-A (β = −0.37, 95%CI from −0.09 to −0.65, *t* = −2.64, *p* = 0.014) and FCSRT – Delayed free recall (β = 0.26, 95%CI from 0.01 to 0.51, *t* = 2.07, *p* = 0.049), while MMSE score, SCWT and DOT Total did not appear as significant predictors.

Before predicting the *Emotion Recognition Test* (RMET) findings, after removing suppressors, the final list of predictors contained FCSRT-Total free recall, FCSRT – Delayed free recall, ACE-R Verbal Memory, TMT-A, BNT, and ACE-R-Language. Regression model was significant (*F* = 11.088; df = 6.24; *p* = 0.0001) in CD patients, explaining 73.5% of the variance. Standardized beta coefficients show that only BNT – Total (β = 0.612, 95%CI from 0.34 to 0.88, *t* = 4.406, *p* = 0.0001) represents a significant predictor of achievement on RMET in CD patients.

## Discussion

Our study shows that patients with CD (SCA1, SCA2, and ILOCA) exhibit impaired performance on both the *Faux Pas Recognition Test* and the RMET compared with HC. The patients have difficulty understanding the mental states of others in everyday interactions and from their facial expressions.

Our study examined two groups of patients: a group of SCA1 and SCA2 patients with severe cerebellar and extra-cerebellar signs and a group of ILOCA patients with primarily isolated cerebellar degeneration. The poor performance of both groups possibly suggests that a cerebellar pathology alone produces social cognition impairment, as previously shown by Hoche (Hoche et al., 2016). SCA patients achieved the worst results on the social cognition tests used in our study, which may suggest that social cognition also involves extra-cerebellar structures (Van Overwalle et al., 2015).

Cerebellar disorders are a group of subentities with origins in various pathophysiological mechanisms; they are also characterized by different levels of social cognitive impairment. Some studies suggest that patients with a left lateral cerebellar tumor (Sokolov et al., 2010) and patients with ponto-cerebellar ischemia (Roldan Gerschcovich et al., 2011) can also exhibit social cognition impairment. Schizophrenia and autism spectrum disorders are further characterized by dysfunctions of cerebellar-cortical networks and an impairment of the “mentalizing” process (Andreasen and Pierson, 2008; Penn et al., 2008; Green et al., 2015).

The duration of the disease was similar in all the patients who took part in our study. The SCA group consisted of younger patients with a more severe clinical presentation and disability (SARA), exhibiting a more serious truncal and limbic ataxia and dysarthria before the commencement of the study. The deleterious effect of the disease in SCA patients, corroborated by the neuropshychological tests, was also evident in other domains of cognition. Unlike our ILOCA patients, the SCA group consistently achieved poorer results compared with the HCs in all examined domains. Routine clinical practice may not have detected these deficits if only MMSE tests had been used, as the SCA patients’ group scores were within the prescribed normal values. However, a detailed neuropsychological evaluation and screening tests such as ACE-R can detect a more serious cognitive deficit. The severity of ataxia in all SCA patients positively correlated with the patients’ cognitive deficit (Ma et al., 2014), although some sources suggest that cognitive functions are affected first and have a slower progression than motor dysfunctions (Fancellu et al., 2013).

Compared to healthy subjects, our SCA patients exhibited consistently poorer results on tests measuring attention and verbal memory than ILOCA patients, which other authors have also previously recorded (Bürk et al., 2001; Le Pira et al., 2002; Fancellu et al., 2013). The ILOCA patients exhibited worse results than the HC in most cognitive domains, but verbal memory, speech and visuospatial deficits were less frequent. Finally, the executive functioning of both the ILOCA and the SCA patients was equally impaired, which previous studies have also found (Bürk et al., 1999, 2001; Rentiya et al., 2018), while the differences in visuospatial tasks proved less consistent (Wallesch and Horn, 1990; Schmahmann and Sherman, 1998; Rentiya et al., 2018). The extent of cognitive impairment, especially that of attention and executive functioning, verbal and semantic memory, language and speech competence, may explain the patients’ poor performance on social cognition tests (Haxby et al., 2000; Siegal and Varley, 2002).

The duration of the disease, functional disability and truncal ataxia significantly correlated with the SCA patients’ performance on the *Faux Pas Recognition Test.* SCWT and *Phonemic Fluency Test*, and significantly correlated with the RMET in the SCA and ILOCA group, respectively, but no correlation was determined with verbally presented social situations in the *Faux Pas* stories.

Although prior studies have shown that social cognition impairments are often associated with executive dysfunction (Aboulafia-Brakha et al., 2011), the literature offers conflicting data (Fine et al., 2001; Clausi et al., 2021). Analysis of connectivity suggests that the cerebellar modules active during social cognition are more likely to be involved in socio-cognitive and not executive networks (Van Overwalle et al., 2015).

Depression and depressive symptoms were the most common non-cognitive symptoms in several studies of patients with cerebellar disorders (Leroi et al., 2002; Liszewski et al., 2004). Although the mean scores in both our groups of patients were within the range for the healthy population, significant difference was observed between the CD patients and the HC. Further, several patients in the SCA1 and 2 groups had a score above 14. The ILOCA group’s anxiety and depression scores correlated with the patients’ performance on the RMET. A larger sample of CD patients is necessary to determine the impact of depression on social cognition. It is possible that the patient’s impaired perception hampers the understanding of other people’s mental states.

The predictive value of EFs in the assessment of social cognition was ambiguous. The EF tests we used did not significantly predict the *Faux Pas* test performance in our SCA patients. SCWT showed significant predictive value for the RMET performance in the SCA group, but not in the ILOCA patients. The most significant predictor in all groups and for both tests was the BNT language and speech test. Language and speech, skills inherent to humans, are primarily an instrument for interpersonal communication (Schmahmann, 2019) and involve the comprehension of words, sentences and stories arising from and serving social interaction. It is thus natural that speech proved to be the best predictor of social cognition among our CD patients (Mariën and Beaton, 2014; Mariën et al., 2014). The social cognition impairment in our CD patients involved diminished semantic lexicon. Errors of this segment inevitably cause difficulties in conducting original cognitive functions and normal social functioning.

Our patients with primary cerebellar disease exhibited impairments in the *Faux Pas Recognition Test* and the RMET, which are traditionally considered indicators of social cognition. As mentioned above, the deficit was primarily caused by semantic processing impairment, but the extent of damage in other cognitive domains determined the severity of social deficit. The literature describes the role of the cerebellum in the cognitive deficit of SCA1 and 2 pathologies, which is commonly attributed to extra-cerebellar degeneration and damaged ties primarily between the basal ganglia and the thalamocortical circles (Gilman et al., 1996; Kawai et al., 2009). Other studies agree with our results, which associate social cognition with specific regions of the brain: mesial and orbitofrontal cortex (Amodio and Frith, 2006; Frith and Frith, 2006; Völlm et al., 2006) and mesial temporal cortex (Gallagher and Frith, 2003; Völlm et al., 2006). Furthermore, (Charlton et al., 2009) it has been shown that poor performance on social cognition tests can be associated with changes in the white matter. We support the assertion that mentalizing processes depend on a complex network which connect different regions and involves ties in the brain’s white matter.

It is unclear whether the obtained data give insight into the patients’ overall social abilities or only into routine components necessary for opinion forming. The metric characteristics of the tests, ecological as well as criterion validity, are of special importance in assessing social cognition. Cognition in real social settings is a dynamic, changeable, and context-dependent process. We concur with (Byom and Mutlu, 2013) that the assessment of social cognition in experimental settings may lead to the under- or overestimation of this complex function, resulting in inconsistent results.

In summary, the findings from this study is that social cognition impairment is present in both the SCA 1 + 2 and the ILOCA patients and that the predictors of this impairment are Efs and language. The conclusions of this study are limited due to the small sample of patients and the fact that they were in the advanced stage of the disease, with great physical and cognitive deterioration. The SCA group was in itself heterogeneous and included two somewhat different entities, where the SCA2 group consisted of patients with severe cognitive impairments, even dementia (Bürk et al., 1999). Because of this, the implication that some brain regions contribute to social cognition must be considered with caution. Future studies must avoid the current study’s limitations.

The body of evidence suggesting the existence of cognitive and mentalizing deficits in CD is growing. We hope our study will encourage theories which embrace the possibility of a completely different profile of the deficit within this heterogeneous disorder. Socialization is the core of all human relationships, which is why the implications of impaired social cognition need to be taken seriously when considering treatment options.

Future research should branch into two directions: researching the cognitive changes in CD and determining the profile of some entities of this heterogeneous disorder. Special attention should be dedicated to operationalizing the mentalizing process, determining the main neuropsychological processes and neural correlates involved, and defining adequate assessment instruments.

## Data Availability Statement

The raw data supporting the conclusions of this article will be made available by the authors, without undue reservation.

## Ethics Statement

The studies involving human participants were reviewed and approved by the Ethical Board of Neurology Clinic, Medical Faculty University of Belgrade. The patients/participants provided their written informed consent to participate in this study.

## Author Contributions

OT and ND-M contributed to the conception and design of the study. OT wrote the first draft of the manuscript. DS performed the statistical analysis. MK, AK, ES, B, AM, M, and NG contributed to the organization and data collection. All authors contributed to manuscript revision, read, and approved the submitted version.

## Conflict of Interest

The authors declare that the research was conducted in the absence of any commercial or financial relationships that could be construed as a potential conflict of interest.

## Publisher’s Note

All claims expressed in this article are solely those of the authors and do not necessarily represent those of their affiliated organizations, or those of the publisher, the editors and the reviewers. Any product that may be evaluated in this article, or claim that may be made by its manufacturer, is not guaranteed or endorsed by the publisher.
